# Loss of Kv8.2 in the Mouse Retina Is Associated With Altered One‐Carbon Metabolism

**DOI:** 10.1111/jnc.70420

**Published:** 2026-03-28

**Authors:** Karina Kruth, Sheila A. Baker

**Affiliations:** ^1^ Department of Ophthalmology and Visual Sciences Carver College of Medicine, University of Iowa Iowa Iowa USA; ^2^ Department of Biochemistry and Molecular Biology, Carver College of Medicine University of Iowa Iowa Iowa USA

## Abstract

Photoreceptors are highly energy‐demanding neurons, and disruption of photoreceptor signaling remodels retinal metabolism and contributes to degeneration, yet the pathways underlying these changes remain incompletely defined. Kv8.2 knockout (KO) mice, a model of KCNV2 retinopathy, exhibit impaired photoreceptor ion homeostasis and slow rod degeneration, providing an opportunity to investigate metabolic adaptation during progressive dysfunction. Untargeted metabolomic profiling was performed on retinas from wildtype (WT) and Kv8.2 KO mice at 1 and 13 months of age. Principal component analysis revealed distinct profiles for aged Kv8.2 KO retinas compared with aged WT and young groups, while young WT and KO retinas were metabolically similar. The major changes in aged Kv8.2 KO retinas compared to aged WT were reduced nucleobases and nucleosides while the amino acids homocysteine, methionine, and serine were elevated. These are signature metabolites in one‐carbon metabolism, a metabolic hub influencing nucleotide metabolism, epigenic regulation, and anti‐oxidant defense. Supervised modeling showed that these one‐carbon–related changes emerge early and progress with age in Kv8.2 KO retinas. Together, these findings implicate altered one‐carbon metabolism as a key mechanism in photoreceptor vulnerability and adaptation in slow retinal degeneration.

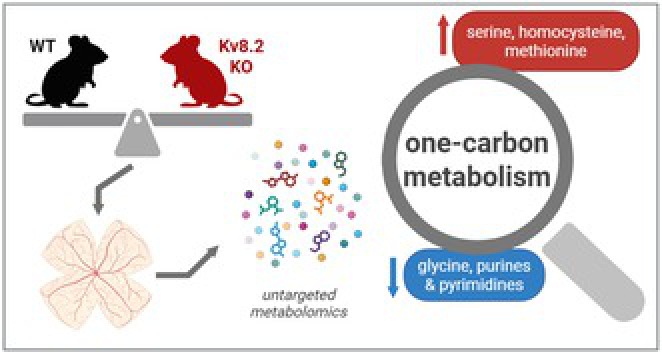

## Graphical abstract

Kv8.2 knockout mice, a model of KCNV2 retinopathy, exhibit impaired potassium homeostasis in photoreceptors and slowly progressive rod degeneration. To investigate metabolic adaptations accompanying this dysfunction, untargeted metabolomic profiling was performed on mouse retinas. The analysis revealed age‐dependent remodeling of retinal metabolism in Kv8.2 knockout mice. Metabolites associated with one‐carbon metabolism, including serine, methionine, and homocysteine, were increased, while nucleotide metabolites derived from purine and pyrimidine synthesis were reduced. These findings indicate that disruption of photoreceptor ion homeostasis is accompanied by reorganization of the one‐carbon metabolic network, linking metabolic adaptation to progressive retinal degeneration.

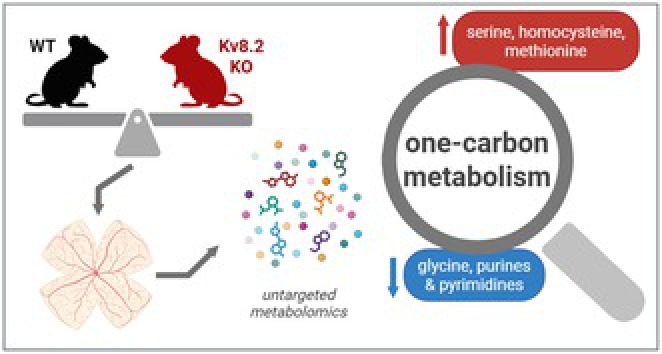

## 1 Introduction

Vision depends on photoreceptors, specialized neurons, whose extraordinary energy demands make them uniquely vulnerable to metabolic disruption (Pan et al. 2021). Because photoreceptors depend on RPE and Müller glia, metabolic changes in disease are not confined to photoreceptors but involve the entire retina. Indeed, profiling studies in animal models show that photoreceptor degeneration remodels the retinal metabolome, yet too few studies exist to define a clear pattern (Xu et al. 2023; Wang et al. 2022; Du et al. 2016; Weiss et al. 2019; Zhou et al. 2021; Xiang et al. 2023; Murenu et al. 2021). We used Kv8.2 knockout (KO) mice, which have impaired photoreceptor ion homeostasis and signaling, along with a moderate loss of rods, to investigate how retinal metabolism adapts to disrupted signaling and slow degeneration.

Kv8.2 is a regulatory subunit of the heteromeric voltage‐gated potassium channel Kv2.1/Kv8.2, expressed in rods and cones (Gayet‐Primo et al. 2018). Studies in animal models (Kv2.1 knockout (KO), Kv8.2 KO, and Kv2.1/Kv8.2 KO mice) demonstrate that the loss of this channel causes the resting membrane potential to be depolarized, resulting in reduced amplitude and delayed signaling (Hart et al. 2019; Jiang et al. 2021; Inamdar et al. 2022; Fortenbach et al. 2021). Electroretinography shows that Kv8.2 KO mice share the same signaling defects observed in patients with the rare inherited retinal disease KCNV2 retinopathy, also known as cone dystrophy with supernormal rod response (Gouras et al. 1983; Guimaraes et al. 2020; Georgiou et al. 2024). In this disease, the abnormal signaling correlates with degeneration of macular cones by the second decade of life, then transitions to a stationary disease as the peripheral retina remains structurally intact in the oldest patients examined (Georgiou et al. 2021; de Guimaraes et al. 2024).

Unsurprisingly, the mouse models of KCNV2 retinopathy have a different pattern of retinal degeneration because mice lack a cone‐enriched macula. In Kv8.2 KO mice, cones remain intact for at least one year (Inamdar et al. 2022). This phenotype suggests that the macular degeneration in KCNV2 patients reflects a feature of the macular microenvironment, rather than an intrinsic vulnerability of cones to the changes in voltage modulation resulting from loss of functional Kv2.1/Kv8.2 channels. It is currently unclear if moderate loss of rods is unique to rodents or a subclinical feature of KCNV2 retinopathy. The degeneration of rods in Kv8.2 KO mice is slow, with 20%–50% outer nuclear thinning occurring between 2 and 12 months of age (Inamdar et al. 2022). In contrast, more commonly studied mouse models of retinal degeneration such as rd1 and P23H have rapid rod degeneration that leaves only ~10% of the ONL by 3 or 9 weeks of age, respectively (Bowes et al. 1990; Chandler et al. 2025; Sakami et al. 2011). Kv8.2 KO mice therefore provide a greater time span to investigate early and adaptive responses to dysfunctional photoreceptors.

By identifying metabolic pathways altered in Kv8.2 KO retina, we aim to clarify mechanisms of photoreceptor vulnerability and adaptation. Our results implicated hyper‐homocysteinema and altered one‐carbon metabolism as a key change in aging Kv8.2 KO retina. One‐carbon metabolism is a biosynthetic hub for nucleotide synthesis, sulfur‐containing amino acids (methionine, homocysteine, cysteine, and taurine), the production of the methyl donor SAM, which is required for epigenetic regulation, and defense against oxidative damage (Ducker and Rabinowitz 2017). All of these processes may critically influence photoreceptor survival and degeneration.

## 2 Materials and Methods

### 2.1 Animals

Kv8.2 knockout (*Kcnv2* ^−/−^, MGI: 7660814) and wild‐type (*Kcnv2* ^+/+^) littermate control mice (congenic on the C57Bl/6J background) were previously characterized (Inamdar et al. 2022). Mice were bred in house; genotyping was performed by Transnetyx (Cordova, TN). Mice were housed in a central vivarium, maintained on a standard 12/12‐h light/dark cycle, with food and water provided ad libitum in accordance with the NIH Guide for the Care and Use of Laboratory Animals. All procedures were approved by the University of Iowa Institutional Animal Care and Use Committee (IACUC) and adhered to the ARVO Statement for the Use of Animals in Ophthalmic and Vision Research.

### 2.2 Metabolite Profiling

For untargeted broad metabolite profiling, each tissue sample consisted of both retinas, ~4 mg of tissue, from a single mouse. Retinas were collected from five wild‐type (WT, 3 male, 2 female) and five Kv8.2 knockout (KO, 2 male, 3 female) mice at 1 month of age, and from four WT and four KO mice at 13 months of age, 2 male and 2 female each group (total *n* = 18) and frozen immediately on dry ice in pre‐chilled plastic tubes. Identification numbers were assigned to individual samples randomly for processing by the Fraternal Order of Eagles Diabetes Research Center Metabolomics Core Facility at the University of Iowa.

Retinal tissue samples were lyophilized overnight, then homogenized using a bead mill in ice‐cold 2:2:1 methanol/acetonitrile/water extraction buffer. The buffer, supplemented with internal standards (D4‐citric acid, D4‐succinic acid, D8‐valine, and U13C‐labeled glutamine, glutamic acid, lysine, methionine, serine, and tryptophan; Cambridge Isotope Laboratories) was used at 90 μL per mg wet tissue weight. Homogenates were rotated for 1 h at −20°C, then centrifuged at 21000×*g* for 10 min. A total of 150 μL of the cleared metabolite extracts was transferred to autosampler vials and dried using a SpeedVac vacuum concentrator (Thermo).

Dried metabolite extracts were reconstituted in 30 μL of 11.4 mg/mL methoxyamine (MOX) in anhydrous pyridine, vortexed for 5 min, and heated at 60°C for 1 h. Then, 20 μL of N,O‐Bis(trimethylsilyl)trifluoroacetamide (TMS) was added to each sample, which was vortexed for 1 min and heated at 60°C for 30 min.

Derivatized samples were analyzed by gas chromatography–mass spectrometry (GC–MS). A 1 μL aliquot was injected into a Trace 1300 GC system fitted with a TraceGold TG‐5SilMS column (Thermo), operating under the following conditions: split ratio = 20:1, split flow = 24 μL/min, purge flow = 5 mL/min, carrier mode = constant flow, and carrier flow rate = 1.2 mL/min. The oven temperature gradient was held at 80°C for 3 min, ramped to 280°C at 20°C/min, and held at 280°C for 8 min. Ion detection was performed by an ISQ 7000 mass spectrometer (Thermo) operated from 3.90 to 21.00 min in EI mode (−70 eV) using select ion monitoring (SIM).

### 2.3 Data Analysis and Visualization

Raw data were analyzed using *TraceFinder 5.1* (Thermo). Metabolite identification required the detection of at least two ions (target and confirming) and a retention time consistent with in‐house reference standards. A pooled‐sample generated prior to derivatization was analyzed at the beginning, at a set interval during, and at the end of the analytical run to correct peak intensities using NOREVA (Li et al. 2017). Corrected data were then normalized to the total signal per sample to control variation in extraction, derivatization efficiency, and sample loading.

Normalized data, available in File S1, were analyzed in *MetaboAnalyst 6.0* without additional transformation or scaling (Pang et al. 2024). Group separation in principal component analysis (PCA) was tested using PERMANOVA. Volcano plots were generated to display log_2_ fold changes versus –log_10_ *p*‐values from unpaired two‐tailed *t*‐tests, with *p*‐values adjusted using the Benjamini‐Hochberg false discovery rate method. sPLS‐DA modeling was computed in R (code available on request).

Generative AI (ChatGPT‐5, OpenAI) was used to assist with R scripting; all final code was reviewed and approved by the authors. BioRender was used to make illustrations of metabolic pathways.

## 3 Results

**FIGURE 1:**
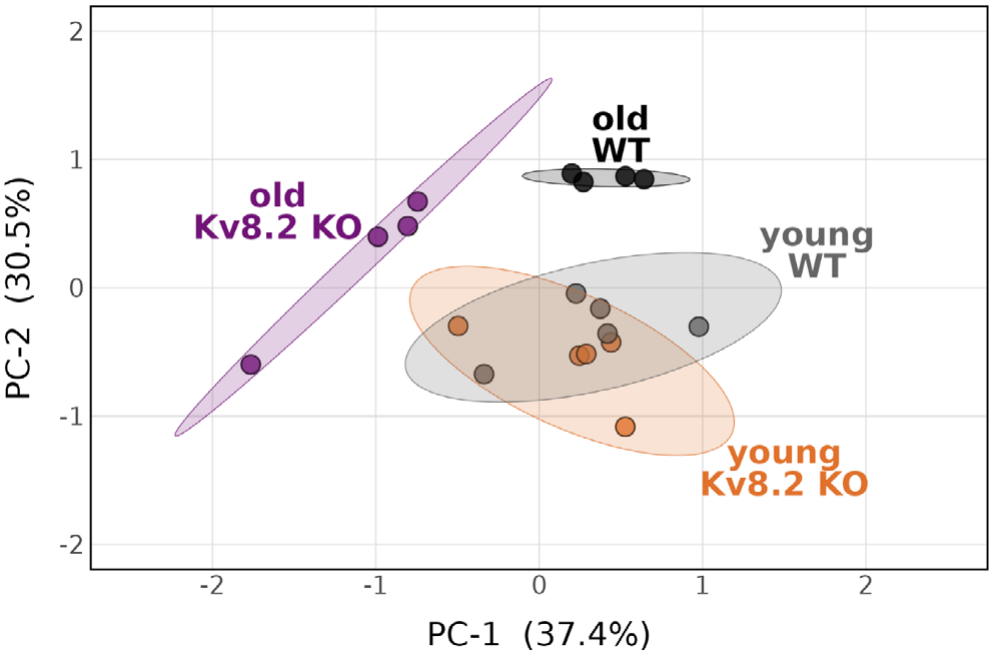
Kv8.2 KO alters the retinal metabolome of aged mice. PCA of normalized metabolite abundance shows four multivariate profiles corresponding to genotype and age. Each point represents one biological replicate, and ellipses represent 95% confidence intervals. Group colors: Dark teal = old (13 months) WT, violet = old (13 months) Kv8.2 KO, teal = young (1 month) WT, and orange = young (1 month) Kv8.2 KO. Separation along PC‐1 (37.4%) and PC‐2 (30.5%) highlights both age‐ and genotype‐dependent metabolic differences.

Untargeted metabolic profiling was carried out on retinas from four experimental groups, WT or Kv8.2 KO at 1 or 13 months of age (young or old). See File S1 for a list of the 101 metabolites that were detected and abbreviations used. PCA demonstrated the data comprised four multivariate profiles (Figure 1). One of the samples in the old Kv8.2 KO group did not cluster as tightly as the others but remained within the 95% confidence ellipse. Sample‐to‐sample variance for the old Kv8.2 and old WT groups is shown as a heat map in File S2. Group separation between old Kv8.2 KO and either old WT or young Kv8.2 KO was significant (*F* = 8.9964, *R* ^2^ = 0.5999, *p*adj = 0.0312 or *F* = 8.5151, *R* ^2^ = 0.5488, *p*adj = 0.0165). The only pairwise comparison that did not show a significant difference was between the young WT and young Kv8.2 KO groups (*F* = 0.3757, *R* ^2^ = 0.0449, *p*adj = 0.6390). The absence of large metabolic differences between young WT and Kv8.2 KO retinas suggests that the effects of Kv8.2 KO emerge with age, prompting further analysis of age‐ and genotype‐dependent metabolic alterations.

**FIGURE 2:**
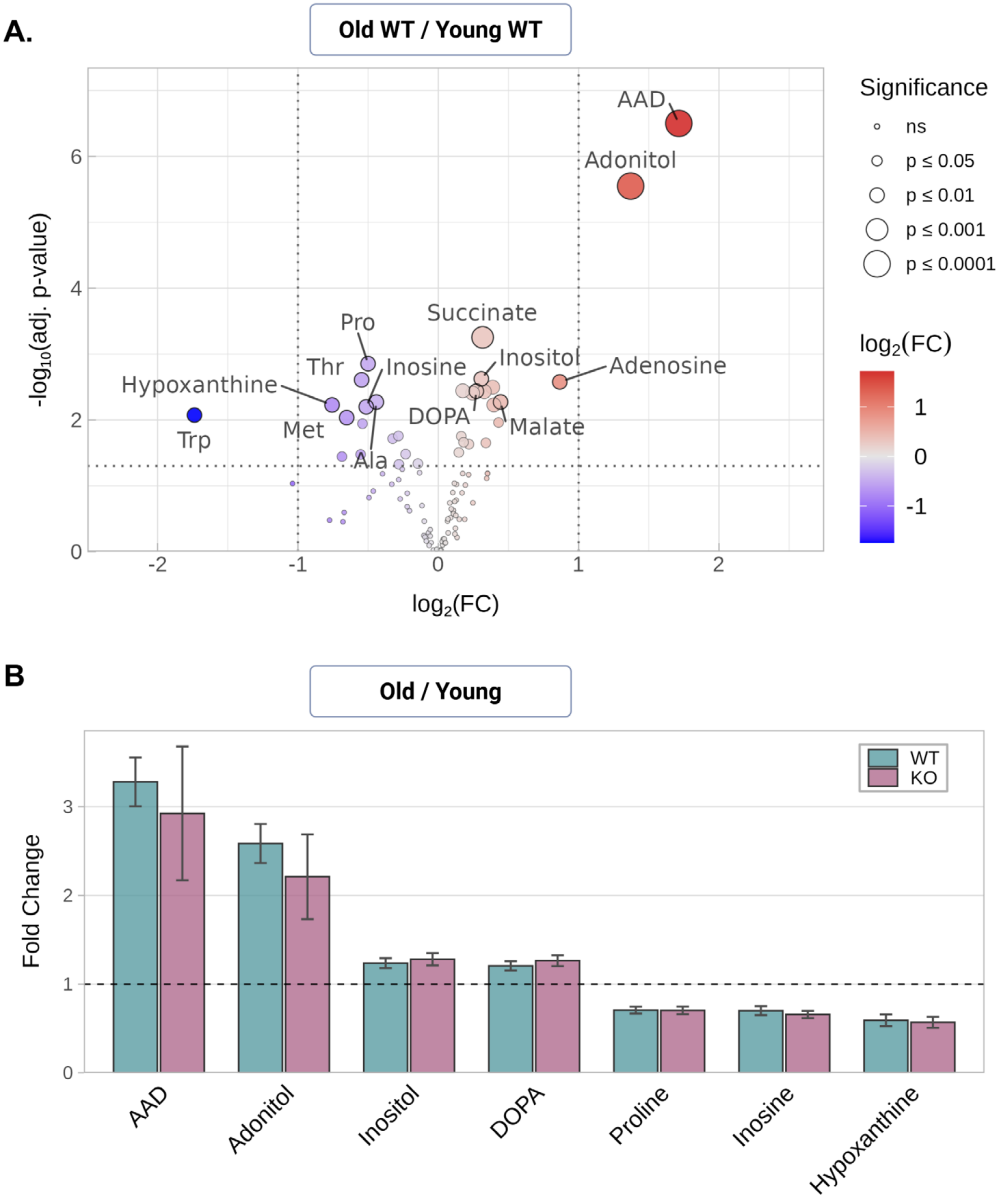
Metabolites altered with age. (A) Volcano plot showing the log_2_(fold change, FC) for individual metabolites in the comparison of old versus young WT groups. Point size and color intensity increase with smaller *p*‐values and greater magnitude of change. Colors indicate direction of change: Red for increased and blue for decreased metabolites in aged WT mice. (B) Correlation of shared metabolites showing significant fold change with age in both WT and Kv8.2 KO. Each point represents the log_2_(FC) for the same metabolite in both genotypes, and the dashed line shows the linear regression fit (*r* = 1.0, *p* = 1.6 × 10^−9^; Pearson correlation), demonstrating that age‐associated alterations are consistent in magnitude and direction between WT and KO.

The aging effect in the different metabolic profiles was examined by comparing the old versus young WT groups (Figure 2A). Several downregulated and about twice as many upregulated metabolites were correlated with aging. The two largest changes were increased levels of aminoadipate (AAD), an intermediate in lysine catabolism, and adonitol (ribitol), the function of which in mammalian tissues is unclear (Huck et al. 2004; Pena et al. 2017). Less significant, there was a change in two purines (increased adenosine and decreased hypoxanthine) and an increase in two of the TCA cycle intermediates (malate and succinate). This is noted because similar changes have been reported in prior metabolic profiling studies of the aging mouse retina (Mu et al. 2024; Wang et al. 2018). The pairwise comparison of old versus young Kv8.2 KO was more challenging to interpret since that data set included both age and genotype dependent effects. However, for the 10 metabolites that showed significant changes in the comparison of old versus young for both genotypes, the log2 fold changes were highly correlated (Pearson, *r* = 1, *p* = 1.6 × 10^−9^); demonstrating that age‐associated alterations were consistent in magnitude and direction between WT and KO (Figure 2B).

**FIGURE 3:**
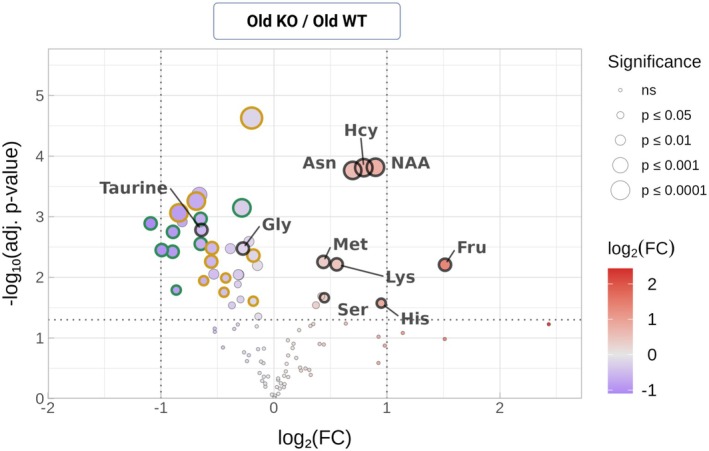
Metabolites altered with genotype in aged retinas. Volcano plot showing the log_2_(FC) for individual metabolites in the comparison of old Kv8.2 KO versus old WT groups. Point size and color represent significance and magnitude of change, respectively, as in Figure 2. Metabolites outlined in green are nucleotide derivatives, and those outlined in dark yellow are fatty acids.

The influence of genotype in the aged retina was assessed by comparing the fold change between old Kv8.2 and the old WT group (Figure 3). The main group of metabolites upregulated in Kv8.2 KO were amino acids (asparagine (Asn), homocysteine (Hcy), n‐acetylaspartate (NAA), methionine (Met), lysine (Lys), serine (Ser), and histidine (His)). There were two classes of metabolites comprising most of the downregulated metabolites: saturated fatty acids and nucleobases/nucleosides. The significance of changes in saturated fatty acids should take account of the ongoing loss of the lipid‐rich photoreceptor outer segments given the degeneration in the aged Kv8.2 KO retina and can only be rigorously interpreted when accompanied by a targeted lipid extraction protocol and analysis which were not done in this study.

**FIGURE 4:**
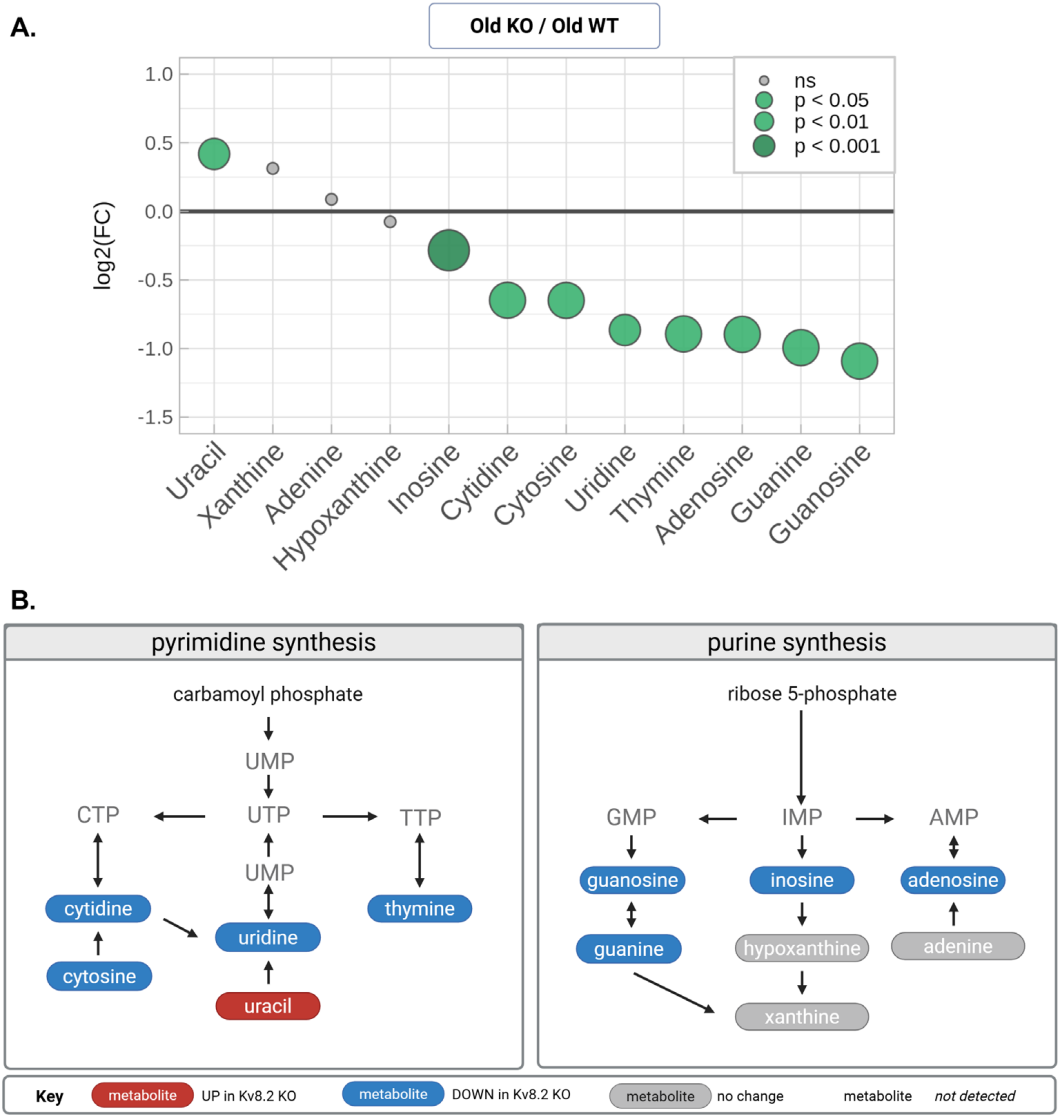
Altered purine and pyrimidine metabolism in Kv8.2 KO retinas. (A) Bubble plot showing the log_2_(FC) for nucleotide metabolites in old Kv8.2 KO versus old WT mice. Bubble size indicates statistical significance (*p*‐value). (B) Schematic diagrams of pyrimidine (left) and purine (right) biosynthetic pathways illustrating relative metabolite changes in Kv8.2 KO retinas. Metabolites decreased in Kv8.2 KO are shown in blue, increased metabolites in red, unchanged in gray, and those not detected are indicated by open symbols.

A closer examination of the nucleobases and nucleosides revealed that both purine and pyrimidine metabolites were affected, with the majority decreased in the Kv8.2 KO relative to WT (Figure 4). This analysis highlights the subset of purine and pyrimidine intermediates that were detected in the profiling dataset and their direction of change, emphasizing a coordinated reduction across multiple points in these pathways. The consistent decrease in purine and pyrimidine intermediates suggests a disturbance in de novo and/or salvage nucleotide synthesis but parsing that out is not possible because, for an unknown reason, we did not capture information about the high‐energy phosphorylated nucleotides. Since nucleotide synthesis is dependent on one‐carbon metabolism, we next turned our attention to the metabolites we detected that are part of that metabolic hub.

**FIGURE 5:**
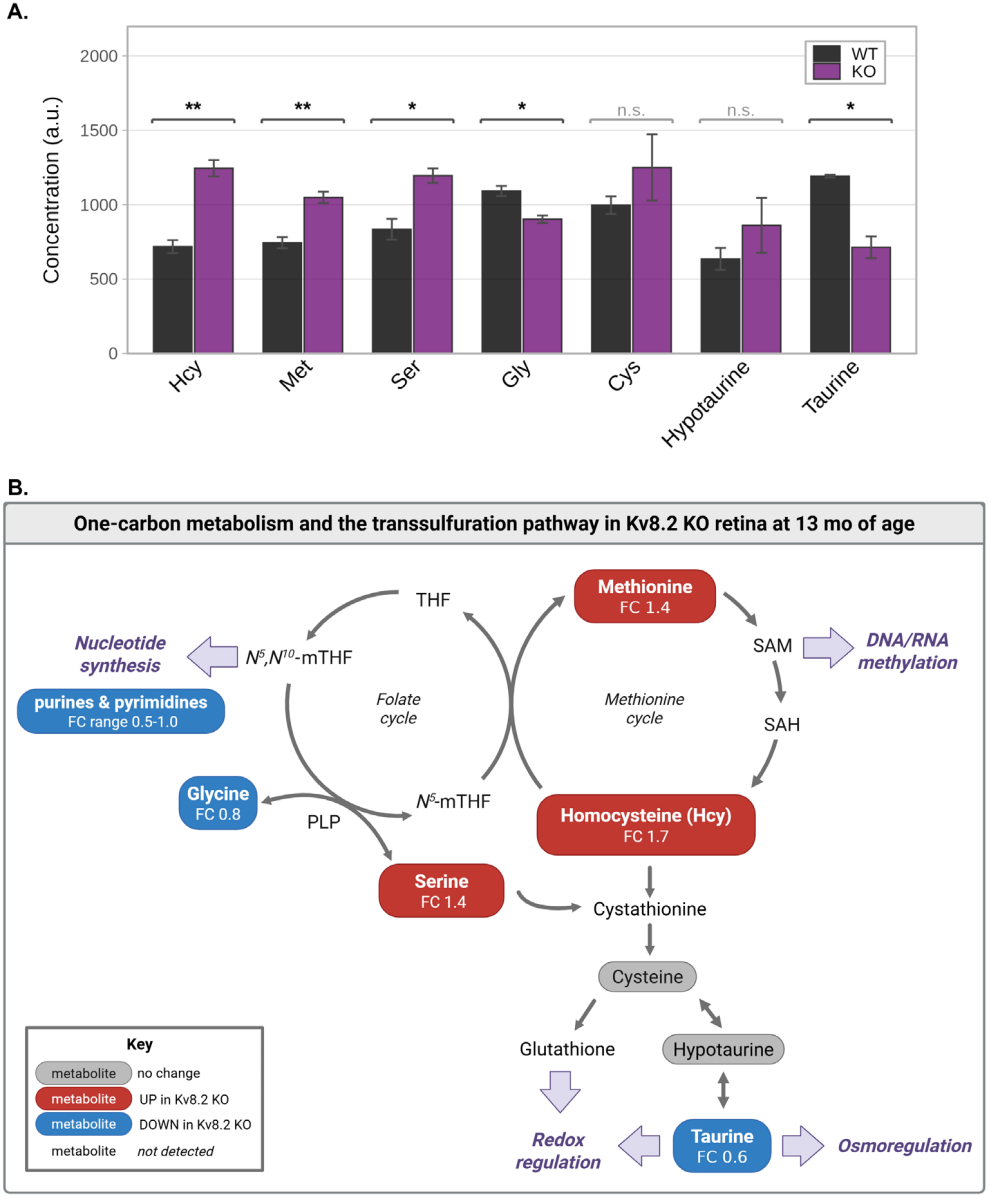
Kv8.2 KO alters one‐carbon metabolism in aged retina. (A) Concentrations of metabolites involved in the one‐carbon and transsulfuration pathways in retinas of old WT and Kv8.2 KO mice. Bars show mean ± SEM; *p* < 0.05, *p < 0.01 (Welch *t*‐test). (B) Schematic representation of one‐carbon metabolism and the transsulfuration pathway in Kv8.2 KO retina. Fold‐change (FC) values indicate metabolite abundance relative to WT. Metabolites increased in Kv8.2 KO are shown in red, decreased in blue, unchanged in gray, and those not detected are shown as open symbols. The accumulation of methionine, serine, and homocysteine (Hcy), together with decreased glycine and taurine, suggests altered flux through the one‐carbon pathway. These changes may influence photoreceptor viability by affecting nucleotide synthesis, epigenetic regulation, and redox or osmotic homeostasis.

Hcy, a key intermediate linking the methionine and cysteine cycles, was significantly elevated in Kv8.2 KO retinas (Figure 5). Increased Hcy was accompanied by higher levels of Met, whereas Cys and hypotaurine showed no significant change, and taurine was decreased. In the interconnected folate cycle, Ser was elevated and Gly reduced, consistent with altered one‐carbon flux. Together, these data indicate that the Kv8.2 KO retina exhibits a coordinated shift in the one‐carbon and transsulfuration pathways, characterized by accumulation of upstream intermediates (Hcy, Met, and Ser).

**FIGURE 6:**
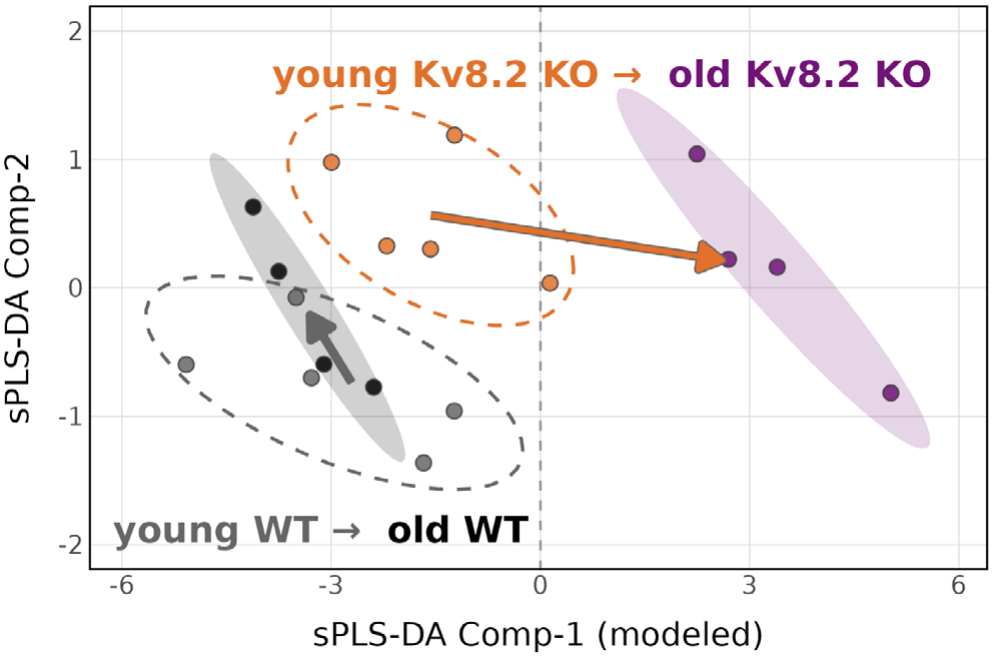
Early divergence of signature metabolites revealed by sPLS‐DA modeling. sPLS‐DA modeling based on signature metabolites differentiating the old WT (teal points with 68% CI shaded ellipse) and old Kv8.2 KO (violet points with 68% CI shaded ellipse) groups. Young WT (light‐teal points) and young Kv8.2 KO (orange points) were projected into the model generated from the old WT and old Kv8.2 KO samples. The young samples drift from their original positions (beginning of colored arrows) toward their respective old clusters. Dashed ellipses indicate the 68% CI for the projected young groups.

To determine whether these metabolic alterations arise early or develop progressively with age, we next applied supervised multivariate modeling to compare the overall metabolomic profiles of young and old WT and Kv8.2 KO retinas (Figure 6). We bioinformatically tested the hypothesis that the key metabolites distinguishing the old WT and old Kv8.2 KO groups would show subtle early changes. The key metabolites selected for this analysis were those related to one‐carbon metabolism—decreased purine and pyrimidine metabolites, glycine, and taurine, and increased serine, homocysteine, and methionine. Using supervised partial least squares–discriminant analysis (sPLS‐DA) modeled on these metabolites, we found that the young WT and young Kv8.2 KO groups were projected to drift toward their respective old clusters (Figure 6). This observation supports the hypothesis that alterations in one‐carbon metabolism emerge early in Kv8.2 KO retinas and may underlie the age‐dependent or degeneration‐associated metabolic divergence observed in the KO.

## 4 Discussion

We report bioinformatic analysis of broad untargeted metabolic profiling on Kv8.2 KO mice. The major implication of this study is that loss of Kv8.2 is associated with altered one‐carbon metabolism, which is a metabolic hub integrating pathways that influence nucleotide availability, epigenic regulation, and the capacity to defend against oxidative. Hindering any of those pathways could compromise photoreceptor viability.

### 4.1 One‐Carbon Metabolism

One‐carbon metabolism can broadly be described as the collection of metabolic reactions involving single‐carbon transfer mediated by folate as the cofactor (Ducker and Rabinowitz 2017). These reactions feed into a larger network of pathways responsible for methionine, serine, glycine, glutathione, S‐adenosylmethionine (SAM), and nucleotide biosynthesis. As a result, perturbations in one‐carbon metabolism affect a wide variety of cellular processes and are implicated in the pathogenesis of neurodegenerative diseases (Lionaki et al. 2022). This includes a range of ocular disorders. For example, high homocysteine levels, as we observed in this study, have been linked to glaucoma, diabetic retinopathy, and age‐related macular degeneration (Tribble et al. 2025; Micheal et al. 2009; Clement et al. 2009; Lei et al. 2018, 2026; Kowluru et al. 2020; Huang et al. 2015).

A striking feature of the aged Kv8.2 KO retina was the coordinated reduction in multiple purine and pyrimidine intermediates, suggesting a broad disturbance in nucleotide homeostasis rather than a block at a single enzymatic step. Nucleotide homeostasis is tightly linked to one‐carbon metabolism, which includes glycine, serine, and the folate cycle, providing key substrates or cofactors for nucleotide synthesis. Either reduced availability or altered utilization of one‐carbon units can lead to diminished nucleotide pools even in the absence of overt energetic failure.

The potential consequences of nucleotide deficiency in long‐lived, post‐mitotic photoreceptors include limited DNA repair from oxidative damage, insufficient RNA for transcription and translation, compromised mitochondrial turnover, and decreased cyclic nucleotides required for signaling. A chronic reduction in nucleotide availability could therefore limit the capacity for cellular maintenance and repair, amplifying the ionic stress resulting from the loss of the outward K^+^ current carried by Kv2.1/Kv8.2 channels in Kv8.2 KO photoreceptors.

Another key aspect of one‐carbon metabolism is the methionine cycle, which generates the major methyl donor SAM. Sustained alterations in this pathway could influence epigenetic regulation of the genome and thereby contribute to long‐term metabolic or transcriptional adaptation in aging Kv8.2 KO photoreceptors. Future transcriptomic analyses of Kv8.2 KO retina may help identify protective or compensatory pathways associated with altered one‐carbon metabolism.

Importantly, supervised multivariate modeling suggests that these alterations in one‐carbon–related metabolites are not restricted to advanced disease stages. When the metabolomic profiles were modeled using one‐carbon–associated metabolites, young Kv8.2 KO retinas were projected toward the aged Kv8.2 KO metabolic state, despite minimal separation by unsupervised analysis at 1 month of age. This finding indicates that disruption of one‐carbon metabolism likely emerges early and progresses over time, preceding substantial photoreceptor loss. Such early metabolic remodeling is consistent with an adaptive response to chronic photoreceptor dysfunction rather than a passive consequence of degeneration.

### 4.2 Amino Acids Upregulated in Kv8.2 KO

Of the seven amino acids increased in Kv8.2 KO, four (Lys, His, NAA, and Asn) are not directly part of the one‐carbon and transsulfuration pathways, but three of those (His, NAA, and Asn) could have indirect ties. For instance, His is an amino acid with direct antioxidant properties like Met and taurine, so His levels could increase if taurine and glutathione production from cysteine becomes limiting (Yu et al. 2007; Castelli et al. 2021; Atmaca 2004; Katayama and Mine 2007; Son et al. 2005). Alternatively, the increased levels of His and the other amino acids could be the consequence of increased autophagy stemming either from a need for more fuel substrates to produce energy or as a process activated to help destroy and remove improperly functioning photoreceptors.

### 4.3 Amino Acids Upregulated in Kv8.2 KO—N‐Acetylaspartate

NAA was significantly increased in the aged Kv8.2 KO retina. NAA is abundant in the brain and its loss is used as a biomarker of neurodegeneration (Schuff et al. 2006). NAA is best known for its role in carrying acetyl groups from neuronal mitochondria to oligodendrocytes to fuel myelin synthesis (Moffett et al. 2007; Baslow 2003). Increases in NAA, as we saw here, are a known consequence of the neurodegenerative disorder, Canavan Disease (CD) characterized by the loss of white matter. CD is caused by loss of function mutations in aspartoacylase, the enzyme that hydrolyzes NAA in oligodendrocytes, and NAA levels build up in the absence of the ability to remove the acetate for use in making myelin (Gronbaek‐Thygesen and Hartmann‐Petersen 2024). The axons in the retina are not myelinated, yet there is a role for NAA in the retina, an idea supported by the report of a CD patient also presenting with retinal degeneration (Benson et al. 2021).

We consider three potential interpretations for the accumulation of NAA we observed in Kv8.2 KO retina. (1) It could indicate energetic stress and use of a controversial “mini‐Krebs” cycle in which carbons from glutamate get oxidized by entering the cycle as α‐ketoglutarate, a reaction that produces Asp which is subsequently acetylated (Chen et al. 2024). (2) It could reflect excess acetate buildup, perhaps from degradation of lipids. Though if that were the case, one might expect the accumulation of other acetylated metabolites. We detected two other acetylated amino acids in our profiling, NAG and NAT, and neither changed in abundance in the Kv8.2 KO retina. (3) The increased NAA could be functioning as an organic osmolyte (Baslow 2003; Warepam et al. 2020). Organic osmolytes like taurine, NAA, betaine, and myo‐inositol function in maintaining cellular volume homeostasis (Pasantes‐Morales 2017; Pasantes‐Morales and Cruz‐Rangel 2010). NAA is especially abundant in the brain. In the retina taurine, a free amino acid synthesized from cysteine, is the most abundant osmolyte (Pasantes‐Morales 2017; Lima et al. 2004; Heinamaki et al. 1986). Since we observed a decrease in taurine levels in the Kv8.2 KO retina, NAA levels may increase in compensation for loss of taurine.

### 4.4 Amino Acids Upregulated in Kv8.2 KO—Asparagine

Asn was significantly upregulated, similar to Hcy and NAA in the aged Kv8.2 KO retina yet is a metabolite not directly related to either of those amino acids. Asn is primarily thought to be simply a substrate for protein synthesis. However, asparagine levels may be playing a role in retinal adaptation to slow degeneration through its function as an amino acid exchange factor. Specifically, asparagine levels have been shown to regulate uptake of serine, arginine, and histidine, with the effect on serine being the most pronounced. Through this mechanism, asparagine has been shown to regulate mTORC1 activity, thereby influencing downstream functions, including nucleotide and protein synthesis, nutrient sensing, and cell growth. Photoreceptors are not replicating but are constantly growing outer segments and, as in all retinal degenerations, Muller glia are activated so it may be worth testing for ties between asparagine and serine in neural retina (Krall et al. 2016).

### 4.5 Limitations

The major limitation of this study is the very nature of broad metabolic profiling. Metabolites are highly labile, and the profiling captures a single snapshot in time of relatively few metabolites. We provide a theoretical framework connecting the changes we observed in the aged Kv8.2 KO retina while recognizing that subsequent deep profiling and flux analysis of key metabolites in one‐carbon metabolism will be necessary to validate the observations made here. Investigating additional Kv8.2 KO mouse ages to precisely define the onset of the signature metabolite changes observed here would be helpful to determine the correlation between metabolic remodeling and the amount of retinal degeneration. Another major limitation is the lack of spatial information provided by broad metabolic profiling. Photoreceptors are the most numerous cells in the retina, and Kv2.1/Kv8.2 channels are only expressed in photoreceptors, so it is reasonable to expect that many of the observed altered metabolites reflect changes in photoreceptors. However, photoreceptors cannot survive on their own; they are part of an interdependent metabolic ecosystem that minimally includes the RPE and Müller glia. Müller glia span the entire retina with processes threaded among the various layers, making it difficult to isolate changes stemming from photoreceptors, Müller glia, or both. However, the RPE can be dissected separately from the retina, and insight could be gleaned by testing for metabolic adaptations within the RPE to the altered physiology of Kv8.2 KO photoreceptors.

Despite these limitations, this study implicates altered one‐carbon metabolism and nucleotide deficiency in the course of slow retinal degeneration due to loss of Kv8.2. The simplest way to influence one‐carbon metabolism is through folate or methylfolate supplementation, which can accelerate flux through all related one‐carbon pathways. Indeed, several studies have found that folate supplementation may be protective in various vision‐related diseases, including retinal vascular diseases, diabetic retinopathy, retinopathy of prematurity, and glaucoma (Lee et al. 2024; Kang et al. 2014; Gu et al. 2023; Malaguarnera et al. 2015; Lei et al. 2019; Nian et al. 2025). Our observations support future research testing if folate supplementation is protective in KCNV2 retinopathy.

## Author Contributions

**Karina Kruth:** writing – original draft, visualization, formal analysis. **Sheila A. Baker:** conceptualization, investigation, funding acquisition, writing – original draft, methodology, validation, visualization, writing – review and editing, formal analysis, software, project administration.

## Funding

National Eye Institute (R01EY020542) and Foundation Fighting Blindness (BR‐CMM‐0619‐0763‐UIA).

## Disclosure

Statement on Use of Generative AI: ChatGPT‐5, (OpenAI) was used to provide recommendations on draft text to improve clarity and flow. All authors edited and approved the final manuscript.

## Conflicts of Interest

The authors declare no conflicts of interest.

## Supporting information


**File S1:** jnc70420‐sup‐0001‐Supplementary File S1.xlsx.

**File S2:** (related to Figures 1 and 3): Heat map of sample variance for old WT (left 4 columns) and old Kv8.2 KO (right 4 columns).

## Data Availability

The normalized metabolite amounts are provided in File S1 and the R code used for sPLS‐DA modeling is available on request.
