# Supplementary material for: Loss of Kv8.2 in the Mouse Retina Is Associated With Altered One‐Carbon Metabolism
Source: J Neurochem. 2026 Mar 28;170(4):e70420. doi: 10.1111/jnc.70420 (PMC13032052; doi:10.1111/jnc.70420)
Supplement: Supplementary file 2 — File S2: (related to Figures 1 and 3): Heat map of sample variance for old WT (left 4 columns) and old Kv8.2 KO (right 4 columns). [file JNC-170-e70420-s001.pdf]

Loss of Kv8.2 in the mouse retina is associated with altered one-carbon metabolism.

Karina Kruth and Sheila A. Baker

SUPPLEMENT

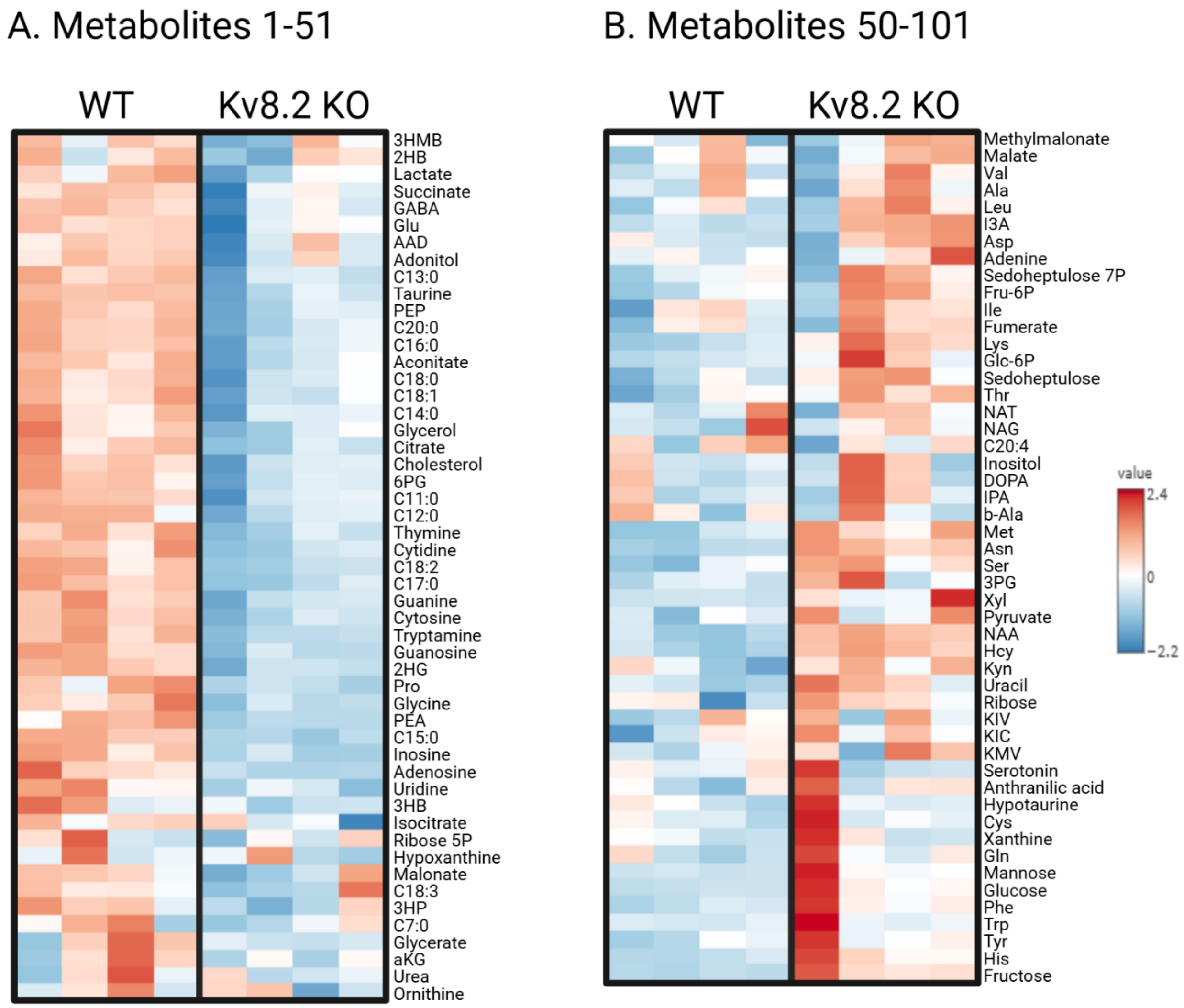

Supplementary File S2 (related to Figures 1 and 3): Heat map of sample variance for old WT (left 4 columns) and old Kv8.2 KO (right 4 columns).
